# Changes in AXL and/or MITF melanoma subpopulations in patients receiving immunotherapy

**DOI:** 10.1016/j.iotech.2024.101009

**Published:** 2024-11-15

**Authors:** M. Willemsen, J. Bulgarelli, S.K. Chauhan, R.R. Lereim, D. Angeli, G. Grisendi, G. Krebbers, I. Davidson, J.A. Kyte, M. Guidoboni, R.M. Luiten, W.J. Bakker

**Affiliations:** 1 Department of Dermatology and Netherlands Institute for Pigment Disorders, Amsterdam University Medical Centers, Location AMC, University of Amsterdam, Cancer Center Amsterdam, Amsterdam Institute for Immunology and Infectious Diseases, Amsterdam, The Netherlands; 2 Immunotherapy Cell Therapy and Biobank (ITCB) Unit, IRCCS Istituto Romagnolo per lo Studio dei Tumori (IRST) “Dino Amadori”, Meldola, Italy; 3 Department of Cancer Immunology, Institute for Cancer Research, Oslo University Hospital, Oslo, Norway; 4 Unit of Biostatistics and Clinical Trials, IRCCS Istituto Romagnolo per lo Studio dei Tumori (IRST) “Dino Amadori”, Meldola, Italy; 5 Laboratory of Cellular Therapy, Division of Oncology, Department of Medical and Surgical Sciences for Children & Adults, University of Modena and Reggio Emilia, Modena, Italy; 6 Department of Functional Genomics and Cancer, IGBMC, CNRS/INSERM, Illkirch, France; 7 Department of Clinical Cancer Research, Oslo University Hospital, Oslo, Norway; 8 Department of Oncology, Ferrara University Hospital, University of Ferrara, Ferrara, Italy

**Keywords:** melanoma, AXL, MITF, DC vaccination, immune checkpoint, tumor heterogeneity

## Abstract

**Background:**

Tumor heterogeneity is a hurdle to effective therapy, as illustrated by the ‘mixed responses’ frequently seen in immunotherapy-treated patients. Previously, AXL+ tumor cells were identified to be highly resistant to targeted therapy, whereas more differentiated MITF+ tumor cells do respond to RAF and MEK inhibitors.

**Patients and methods:**

In this study, we analyzed tumor heterogeneity and explored the presence of the previously described AXL+ or MITF+ melanoma subpopulations in metastatic tissues by NanoString gene expression analysis, single-cell RNA sequencing and *in situ* multiplex immunofluorescence. Furthermore, we analyzed how these subpopulations correlate with immunological pressure and response to immunotherapy by immunomodulating antibodies or autologous tumor lysate-loaded dendritic cell vaccination.

**Results:**

Our data demonstrate large interpatient variability and variable therapy-induced changes independent of the type of therapy. We identify the presence of previously described AXL+ and MITF+ subpopulations in metastatic tissues both at the mRNA level and *in situ* at the protein level, and demonstrate that MITF+ melanoma cells are significantly decreased upon immunotherapy, while AXL+ melanoma cell numbers are stable. MITF+ tumor cells showed the most significant inverse correlation with CD8+ T cells. Our patient cohort also shows that immunotherapy-induced changes in the abundance of AXL+ or MITF+ tumor cells did not correlate with improved survival.

**Conclusions:**

Overall, this study suggests that more differentiated MITF+ tumors are efficiently targeted by immunotherapy, while AXL+ tumor cells may be more resistant, analogous to their response to targeted therapy.

## Highlights

• Tumor heterogeneity is a hurdle to effective therapy, illustrated by the resistance of AXL+ tumor cells to targeted therapy.
• Here we analyzed the changes of AXL+ and/or MITF+ subpopulations in response to immunotherapy in metastatic melanoma.
• AXL+ and/or MITF+ melanoma subpopulations were analyzed by NanoString, scRNAseq and multiplex immunofluorescence.
• MITF+ melanoma subpopulations significantly decreased upon immunotherapy, and inversely correlated with T cell infiltration.
• Changes in the abundance of AXL+ and/or MITF+ subpopulations did not correlate with patient survival.
• Our study shows that MITF+ melanoma subpopulations, are more efficiently targeted by immunotherapy than AXL+ subpopulations.

## Introduction

Melanoma is one of the most aggressive cancers, with an extraordinary ability to metastatically spread both at regional lymph nodes and at distant sites. Although melanoma patients generally respond well to immunotherapy, a significant fraction of patients do not benefit.^1^ In addition, in some patients secondary resistance may occur and it is not unusual observing ‘mixed responses’, with some tumor lesions regressing and others progressing,^2^ which is at least in part due to tumor heterogeneity.

Intrapatient tumor heterogeneity involves the presence of tumor cells with different genetic and phenotypic features within a tumor lesion (intralesional) or between different tumor lesions (interlesional). Resistance to immunotherapy might result from selective outgrowth of antigen-negative tumor cells^3^, ^4^, ^5^, ^6^ or from tumor cells with stemness properties that phenotypically differ from their more differentiated counterparts.^7^^,^^8^ Earlier studies identified melanoma cells from two main distinct transcriptional cell states based on the expression of the MITF transcription factor, which drives the gene expression program of differentiated melanocytic-type cells, or the receptor tyrosine kinase AXL, a marker of undifferentiated mesenchymal-type cells.^9^, ^10^, ^11^ Targeted therapy with BRAF/MEK inhibitors has effect against BRAF-mutated melanoma.^12^ In contrast to MITF^high^ (MITF+) melanoma cells, which in general are responsive to RAF and MEK inhibitors, melanoma cells expressing high levels of AXL are associated with resistance.^9^^,^^10^^,^^13^ Resistance to targeted therapy can thus result from the selection of tumor cells lacking target expression or from up-regulation of rescuing molecules or pathways (e.g. multidrug resistance proteins, WNT signaling^14^ or up-regulation of alternative tyrosine kinase receptor such as AXL^10^^,^^15^). Immunotherapy by checkpoint inhibitors or whole tumor vaccines might be less affected by this type of resistance, although patients who show acquired resistance to anti-mitogen-activated protein kinases targeted therapy often develop cross-resistance to immunotherapy.^16^^,^^17^ In contrast, resistance to immunotherapy, either primary or secondary, involves several different mechanisms that lead to immune escape of tumor cells,^18^^,^^19^ e.g. loss of antigen or human leukocyte antigen (HLA) expression,^18^ up-regulation of immune checkpoint molecules other than those targeted,^20^ β-catenin signaling,^14^ phosphatase and tensin homolog loss,^18^^,^^21^ microbiota features^22^ and immune suppressive cells and cytokines.^20^ Interestingly, immunotherapy itself can also directly cause tumor heterogeneity. It has been shown that tumor-specific cytotoxic T cells induce dedifferentiation of melanoma cells, which thereby acquire ‘stem cell-like’ properties.^23^ Tumor cells with stemness features are less immunogenic and are associated with anticancer immunity.^24^ These examples illustrate that due to tumor heterogeneity, melanoma cells can evade immune destruction or targeted therapy and indicate the need to understand melanoma heterogeneity in relation to therapy.

This study aimed to identify distinct melanoma cell subpopulations likely provided with different resistance to immunotherapy, and how they change along different treatments, i.e. dendritic cell (DC) vaccines or ipilimumab. In particular, the response of MITF- and/or AXL-expressing melanoma cells to current immunotherapies was investigated, as well as their association with immunological pressure from tumor-infiltrating lymphocytes. To this end, we analyzed the phenotypically diverse tumor cell subpopulations of metastatic melanoma lesions taken at the baseline and after starting immunotherapy based on differential expression of AXL and MITF evaluated by single-cell RNA sequencing (scRNAseq), gene expression analysis and multiplex immunofluorescence (mIF) *in situ* imaging.

## Patients and methods

### Patient material

Fragments of resected metastases from melanoma patients pre- and post-immunotherapy [autologous tumor lysate-loaded DC vaccination (*n* = 8)^25^ or ipilimumab (*n* = 8), pembrolizumab (*n* = 1), nivolumab (*n* = 2)] were collected by the Department of Immunotherapy, Cell Therapy and Biobank Unit at the IRCCS Istituto Romagnolo per lo Studio dei Tumori (IRST) “Dino Amadori” (Meldola, Italy). Resected tumor material was either processed for scRNAseq purposes and/or was embedded in formalin-fixed paraffin-embedded (FFPE) blocks (Supplementary Figure S1, available at https://doi.org/10.1016/j.iotech.2024.101009). Multiple 4-μm FFPE sections were cut for hematoxylin–eosin (H&E) staining and for protein expression analyses using mIF. One 10-μM section was used for gene expression analysis by NanoString as described below. The study was approved by the CEROM Ethics Committee (approval n° 2639/2019 I.5/63 of 13 March 2019) and was conducted in accordance with the principles laid down in the 1964 Declaration of Helsinki. Written informed consent was obtained from all participants. Detailed patient characteristics are listed in Table 1.

**Table 1:** Patient characteristics and clinical response

Patient ID	Age (years)/sex	Mutation status	Prior treatment	Treatment	Site of tumor resection/months pre- or post-therapy		Following treatment	BOR (RECIST)/duration (months)	Overall survival (months)	Objective response	Tissue analysis		
					Pre-treatment	Post-treatment					scRNAseq	NanoString	mIF
P1	60/M	N/A	None	DC vax	Adrenal gland/2	Adrenal gland/OT	None	SD/6	46.5	Progressing	✖	✓	✓
P2	45/M	V600E	None	DC vax	Lymph node/1	Lymph node/OT	CT, HD IL-2	CR/8	34	Progressing	✖	✖	✓
P3	36/F	V600E	BioCT	DC vax	Omentum/2	Stomach/OT	Surgery, RT	SD/50	122+	Stable	✖	✖	✓
P4	64/M	V600E	BioCT	DC vax	Lymph node/15	Subcutis/18	Surgery	PR/68	87	Stable	✖	✓	✓
P5	68/M	V600E	BioCT	DC vax	Lung/3	Skin/7	LD IL-2	SD/9	62	Progressing	✖	✓	✓
P5 Ipi	69/M	V600E	LD IL-2	Ipi	Skin/6	Subcutis/16	None	PD	37	Progressing	✖	✓	✓
P6	35/F	WT	CT, Ipi	DC vax	Peritoneum/2	Jejunum/OT	RT	SD/4	16	Progressing	✖	✓	✓
P7	31/M	V600E	None	DC vax	Skin/2	Skin/OT	Ipi, Vem, BioCT, Pembro	PD	19	Progressing	✖	✓	✓
P8	79/M	WT	None	Ipi	Skin/1	Subcutis/6.5	CT	PD	10.5	Progressing	✖	✖	✓
P9	44/M	N/A	BioCT	Ipi	Skin/0	Subcutis/26	CT	PD∗	194+	Progressing	✖	✓	✓
P10	45/M	N/A	None	Ipi	Lymph node/0	Lymph node/19	DC vax	PR/17	25.5	Progressing	✖	✓	✓
P11	31/M	V600E	None	DC vax	Lymph node/3	Skin/5.5	Ipi, Vem, CT, Pembro	PD	19	Progressing	✖	✓	✖
P12	58/F	V600E	CT	Ipi	Skin/0.5	Lymph node/0.5	None	PD	1	Progressing	✖	✓	✓
P13	73/F	WT	CT	Ipi	Subcutis/3.5	Lung/2.5	None	PD	3.6	Progressing	✖	✓	✓
P14	65/M	V600E	None	Ipi	Skin/0	Skin/1	None	CR/169+	192+	Stable	✖	✓	✓
P153	89/M	N/A	None	Surgery	Lymph node/0	N/A	None	NED after surgery	—	—	✓	✖	✓
P196	53/M	WT	None	Ipi	N/A	Subcutis/OT	DC vax, Nivo, Tem	PD	20	Progressing	✓	✓	✓
P256	66/F	WT	None	Adjuvant Nivo	Subcutis/1	N/A	None	NED/6	17	—	✓	✓	✓
P258	41/M	WT	Ipi	Pembro	N/A	Subcutis/4.5	HD IL-2	PD	3	—	✓	✓	✓
P262	45/F	V600E	None	Adjuvant Nivo	Lymph node/2.5	N/A	None	NED/13	13	—	✓	✓	✓

—, missing data; BioCT, cytokine (IL-2 and/or IFN-α) + chemotherapy; BOR, best overall response; CR, complete response; CT, chemotherapy; DC vax, dendritic cell vaccination; F, female; IFN-α, interferon-α; IL-2, interleukin 2; Ipi, ipilimumab; M, male; mIF, multiplex immunofluorescence; N/A, not available; NED, no evidence of disease; Nivo, nivolumab; OT, on treatment; PD, progressive disease; Pembro, pembrolizumab; PR, partial response; RT, radiotherapy; scRNAseq, single-cell RNA sequencing; SD, stable disease; Tem, temozolomide; Vem, vemurafenib; WT, wild type.

### Gene expression analysis [NanoString nCounter Technology (NanoString)]

**Figure 1:**
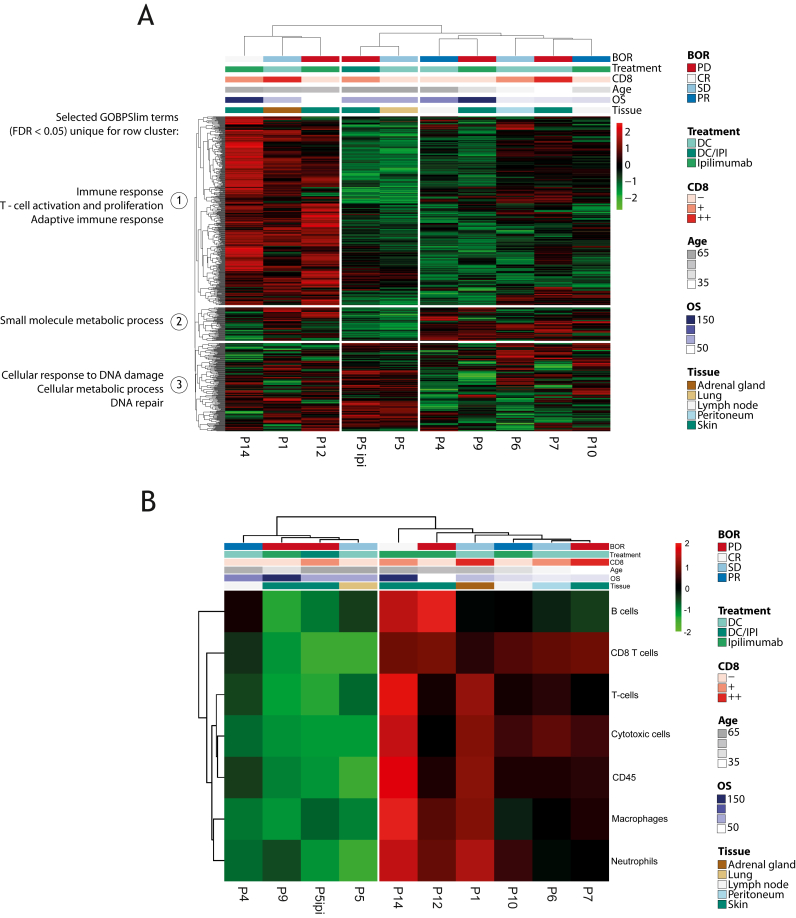
**NanoString analysis identifies phenotypic heterogeneity among patients.** (A) Heatmap showing relative gene expression across the pre-immunotherapy melanoma tumor samples, as determined by NanoString IO360 gene expression assays. Selected gene ontology biological processes differentially enriched in the patient clusters using gene set enrichment analysis are shown on the left. Selected annotations (BOR, treatment, CD8 infiltration determined by IHC, age, OS and tissue origin) are shown on the top. (B) Heatmap representing the relative proportion of immune cells, as determined by NanoString gene signatures. NanoString data (A-B) show samples from patients that were also analyzed by mIF (Figure 3B). BOR, best overall response; CR, complete response; DC, dendritic cell; IHC, immunohistochemistry; IPI, ipilimumab; mIF, multiplex immunofluorescence; OS, overall survival; PD, progressive disease; PR, partial response; SD, stable disease.

The PanCancer IO360 gene expression panel (NanoString Technologies, Seattle, WA) was used to determine the expression of 800 human genes. It combines vital components involved in the complex interplay between the tumor, microenvironment and immune response in cancer. The panel was extended with 30 custom genes: *CCR6*, *CCR9*, *CCR7*, *Clec10a*, *CMTM6*, *RQCD1*, *CXCR1*, *CXCR5*, *ERVK-2*, *ERV3-2*, *FMOD*, *GPNMB*, *IFNB1*, *IL12A*, *IL21*, *IL5*, *IL23A*, *IL9*, *KLRG1*, *Ly6E*, *MAFB*, *MIF*, *PRAME*, *RGS5*, *ROR1*, *SMAD2*, *STEAP1*, *TERT*, *TF* and *TYR*. Tumor RNA was isolated from 10-μm FFPE tissue sections from baseline biopsies of 16 melanoma patients. Briefly, FFPE sections were deparaffinized using (R)-(+)-Limonene (Merck, Darmstadt, Germany, 183164) and absolute ethanol and air-dried for 15 min. Macrodissection was done in a way so only tumor tissue was collected with H&E slide used as a reference. RNA isolation from collected tumor tissue was done using the High Pure FFPET RNA Isolation Kit (Roche, Basel, Switzerland, 06650775001) as per manufacturer’s recommendations. Hybridization of RNA and capture/reporter probes was done using the nCounter XT Assay protocol (NanoString Technologies). Post-hybridization processing was carried out on the nCounter Prep Station as per manufacturer’s recommendations. The nCounter Digital Analyzer was used for digital counting and data collection with field of view set to 555. Preliminary quality control was done using the RCCCollector (NanoString Technologies) tool before further data processing. Sample P13 was excluded after quality control because of low messenger RNA (mRNA) content. P11, P0196, P0256, P0258 and P0262 are not shown in Figure 1 as no paired multiplex immunohistochemistry (IHC) data were available for these samples. Only samples with paired multiplex data (pre- and post-therapy samples) and NanoString analysis are shown in Figure 1.

RCC files were analyzed by nSolver 4.0 (NanoString Technologies), using the Cross RLF and batch calibration pipeline. Briefly, the panel standard included in each run was used to calibrate and compare samples from different batches. The background threshold was set to the mean plus 2 standard deviations of the negative control count, otherwise following the standard pipeline. The normalized log_2_ transformed values and the immune cell quantification from the nSolver advanced analysis mode were exported for further analysis in R (v4.3.1, R Foundation for Statistical Computing, Vienna, Austria). The package pheatmap 1.0.12 was used to generate overview heatmaps of the log_2_ gene expression and immune cell subsets. The immune cell subsets had to have a *P* value <0.05 as determined by nSolver to be included in the analysis. The mean log_2_ abundance of fibroblast signature genes^26^ that were above background level in all samples was used as representative for cancer-associated fibroblasts (CAFs). The average log_2_ abundance of the resulting CAF genes was used: ADAM12, CDH11, COL11A1, COL5A1, COL6A3, COMP, CRABP2, CXCL12, CXCL14, DEPTOR, EGFR, FAP, FMOD, GPC4, INHBA, NID2, OLFML2B, PDGFRB, PLA2G2A, PLOD2, ROR2, SFRP4, STEAP1, TGFB3, THY1, TPM1, TWIST1 and VCAN. Pearson correlation was used to compare immune cell abundance to AXL mRNA; *P* value <0.05 was considered significant.

### Tissue handling for scRNAseq analysis

Resected tumor material (a total of five tumors were resected, Supplementary Table S1, available at https://doi.org/10.1016/j.iotech.2024.101009) was mechanically dissociated using scalpels in RPMI (Lonza Group Ltd, Basel, Switzerland) on ice and by pipetting descending sizes, followed by filtering through a 100-μm nylon strainer (Corning, Glendale, AZ). The suspension was immediately placed on ice and upon centrifugation at 470 *g* at 4°C for 5 min, and the cell pellet was resuspended in phosphate-buffered saline with 2% fetal calf serum. An aliquot was immediately analyzed by fluorescence-activated cell sorting (FACS) analysis and sorted, while the remaining cell suspension was vitally frozen and stored in liquid nitrogen for subsequent use. Gene signatures that were analyzed (Immune and Pigmentation) are provided in Supplementary Table S2, statistical analysis in Supplementary Table S3.

### Cell sorting for scRNAseq analysis

Single-cell suspensions were stained with APC-H7-conjugated mouse anti-human CD45 (clone 2D1, BD Biosciences, Franklin Lakes, NJ) and Calcein AM (Life Technologies, Carlsbad, CA). Firstly, doublets were excluded from the analyses. Then, viable, non-immune (Calcein^high^ CD45−) cells were sorted by FACSAria II cell sorter (BD Biosciences) into two Precise WTA Single Cell Encoding 96-well plates (BD Biosciences) pre-chilled to 4°C. Next, the plates were sealed, vortexed and centrifuged at 1000 *g* at 4°C for 1 min, immediately placed on dry ice and transferred for storage at −80°C as per manufacturer’s recommendations.

### Whole transcriptome amplification and library preparations for scRNAseq analysis

Whole transcriptome amplification (WTA) and library preparations were carried out using the Precise WTA single-cell kit (BD Biosciences), following the manufacturer’s instructions. The 96 samples of a multiwell plate were pooled together and cleaned two times with 0.8× DNA SPRIselect bead-based reagent (Beckman Coulter, Brea, CA). Library quality was assessed with a high-sensitivity DNA chip (Agilent, Santa Clara, CA) and quantified with a high-sensitivity dsDNA Qubit Kit (Life Technologies). Samples were sequenced on an Illumina HiSeq 4000 instrument using 2 × 100 bp paired-end reads.

### scRNAseq and bioinformatics analysis

Sequencing data were uploaded into the Seven Bridges-hosted pipeline, generating molecular index corrected counts for each gene per cell. Seurat R package was used for further analysis.^27^ Genes were retained if MI_detection field is equal to ‘pass’ and unique molecular identifiers are at least five in at least five cells. On the other hand, cells were filtered out according to the following conditions: raw read count is >200 000; there are >1000 expressed genes; the percentage of mitochondrial genes is <30%. A total number of 395 cells were eventually sequenced (Supplementary Table S1, available at https://doi.org/10.1016/j.iotech.2024.101009).

### Data availability statement

scRNA-Seq data are available upon request on the European Genome-phenome Archive (EGA) at the following URL: https://ega-archive.org/ searching for the manuscript title.

Based on the expression of both AXL and MITF signatures,^9^ tumor cells were categorized into four phenotypic groups: AXL+/MITF+; AXL+/MITF−; AXL−/MITF+; AXL−/MITF−. Then, we carried out differential gene expression analysis between cells belonging to each one of these phenotypic groups against cells of the other phenotypic groups and extracted the top 10 most and the top 10 least expressed genes from each comparison.

### R2 genomics analysis and visualization platform

The R2 platform (http://r2.amc.nl) was used to analyze the cutaneous melanoma dataset from The Cancer Genome Atlas^28^ and a recently published scRNAseq melanoma dataset.^26^

### Multiplex immunofluorescence

mIF staining was carried out according to manufacturers’ instructions (except for antibody removal) using the OPAL seven-color immunohistochemistry kit (Akoya Biosciences, Marlborough, MA). Antibodies used included mouse anti-Melan-A (clone A103, Dako/Agilent, Santa Clara, CA), mouse anti-melanosome (clone HMB-45, Dako/Agilent), mouse anti-tyrosinase (clone T311, Dako/Agilent), mouse anti-MITF (clone D5, Dako/Agilent), mouse anti-CD8 (clone C8/144B, Dako/Agilent), mouse anti-CD45 (clone 2B11+PD7/26, Dako/Agilent), rabbit anti-PRAME (clone EPR20330, Abcam, Cambridge, UK) and rabbit anti-AXL (clone EPR19880, Abcam). Antibody removal was done by β-mercaptoethanol-containing stripping buffer pH 7.5 (2% sodium dodecyl sulfate/Tris-HCl, 0.7% β-mercaptoethanol) for 30 min at 50°C. Slides were mounted with ProLong Diamond Antifade Mountant (ThermoFisher Scientific, Waltham, MA).

### Imaging

The Vectra Polaris Automated Quantitative Pathology Imaging System (Akoya Biosciences) was used for multispectral imaging at ×20. Thereafter, whole slide images were loaded into Phenochart Whole Slide Viewer and InForm image analysis software (both Akoya Biosciences) for unmixing. Component images of 10 high-power fields (5 from the border and 5 from the center of the tumor) were analyzed for phenotyping melanoma subsets and T-cell infiltration in QuPath software (open source software).^29^

## Results

### NanoString analysis of metastatic melanoma samples

To analyze tumor heterogeneity and distinct melanoma cell subpopulations, as well as their changes upon immunotherapy, we investigated both mRNA and protein expression in metastatic tumor samples (Supplementary Figure S1, available at https://doi.org/10.1016/j.iotech.2024.101009). We carried out NanoString gene expression analysis using baseline melanoma samples before immunotherapy (Figure 1A and B). The NanoString analysis revealed large interpatient variability when clustered by gene expression (Figure 1A) or immune cell signatures (Figure 1B). Three patients showed marked up-regulation of gene ontology biological processes (GOBPSlim) terms associated with immune profiles (Figure 1A). Two of these patients had tumor infiltration of CD8+ T cells as assessed by IHC. Analysis of specific immune cell types using gene signatures revealed an overall absence or presence of various immune cell populations in samples, but no enrichment of specific immune cells or an association with response or survival (Figure 1B). As lymphocyte tumor infiltration is associated with improved survival,^28^ and a better response to checkpoint inhibition in melanoma,^30^ the absence of these associations is likely due to our cohort size. The other non-immune baseline gene expression profiles were not associated with best overall response, overall survival (OS) or tumor metastasis location either (Figure 1A).

### Single-cell RNA sequencing identifies distinct transcriptional signatures

**Figure 2:**
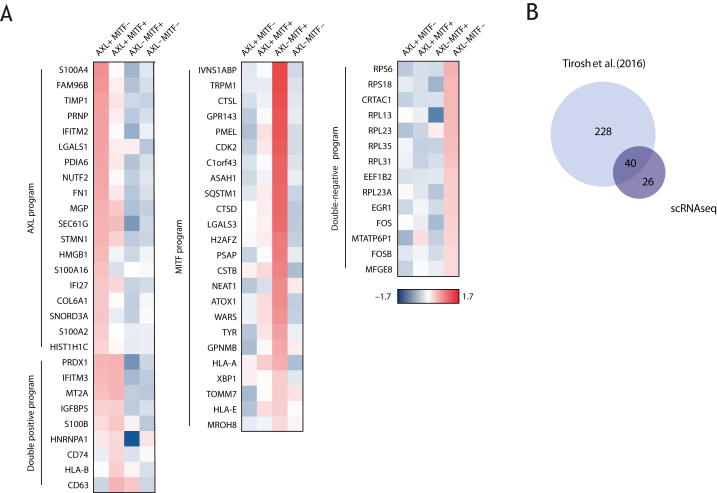
**scRNAseq identifies distinct transcriptional signatures.** (A) Heatmap showing the 66 most differentially expressed genes (population averages) for AXL+ MITF−, AXL+ MITF+, AXL− MITF+ and AXL− MITF− melanoma subpopulations. (B) Venn diagram showing the overlap between the top 10 most differential expressed genes for every subpopulation from the Tirosh et al.^9^ (2016) dataset and the 66 most differentially expressed genes from our scRNAseq analysis. scRNAseq, single-cell RNA sequencing.

We next carried out scRNAseq analysis on a number of fresh tumor samples from which 395 cells were sequenced (Supplementary Table S1 and Figure S2, available at https://doi.org/10.1016/j.iotech.2024.101009) to confirm the presence of the previously identified AXL and MITF melanoma subpopulations (Tirosh et al.^9^). Our scRNAseq data confirmed the presence of at least two distinct melanoma transcriptional signatures identified by the expression of AXL and MITF (Figure 2A). We also identified cells positive (and negative) for both transcriptional programs. From the differential gene expression analysis comparing each group with the others, the top 10 positive and negative genes were identified, generating a list of 120 genes. After excluding duplicate genes from this list, 66 unique genes remain (Figure 2A). *In silico* analysis on the scRNAseq dataset published by Tirosh et al.^9^ revealed similar and additional differentially expressed genes between the subpopulations (Figure 2B). Among the 66 most differentially expressed genes as identified from our scRNAseq analysis, 61% overlapped with those identified by the existing database (Figure 2B).

The AXL+ MITF− cluster was associated with expression of markers of epithelial-to-mesenchymal transition, invasion and metastasis (Figure 2A). The AXL− MITF+ subpopulation was enriched for markers of melanocyte differentiation and pigmentation such as PMEL, TYR and GPR143 (Figure 2A), consistent with MITF driving differentiation of the melanocytic lineage.^31^ Cells expressing both AXL and MITF exhibited increased levels of markers involved in, for example, antigen presentation, including CD74 and HLA-B or HLA-A (Figure 2A). Finally, cells lacking expression of AXL and MITF were characterized by the expression of various ribosomal proteins (Figure 2A), which might reflect Myc activation.^26^^,^^32^ Together, these confirm the presence of the AXL and MITF subpopulations in our patient cohort, allowing the analysis of their responsiveness to immunotherapy.

### Melanoma composition shows marked interpatient heterogeneity

**Figure 3:**
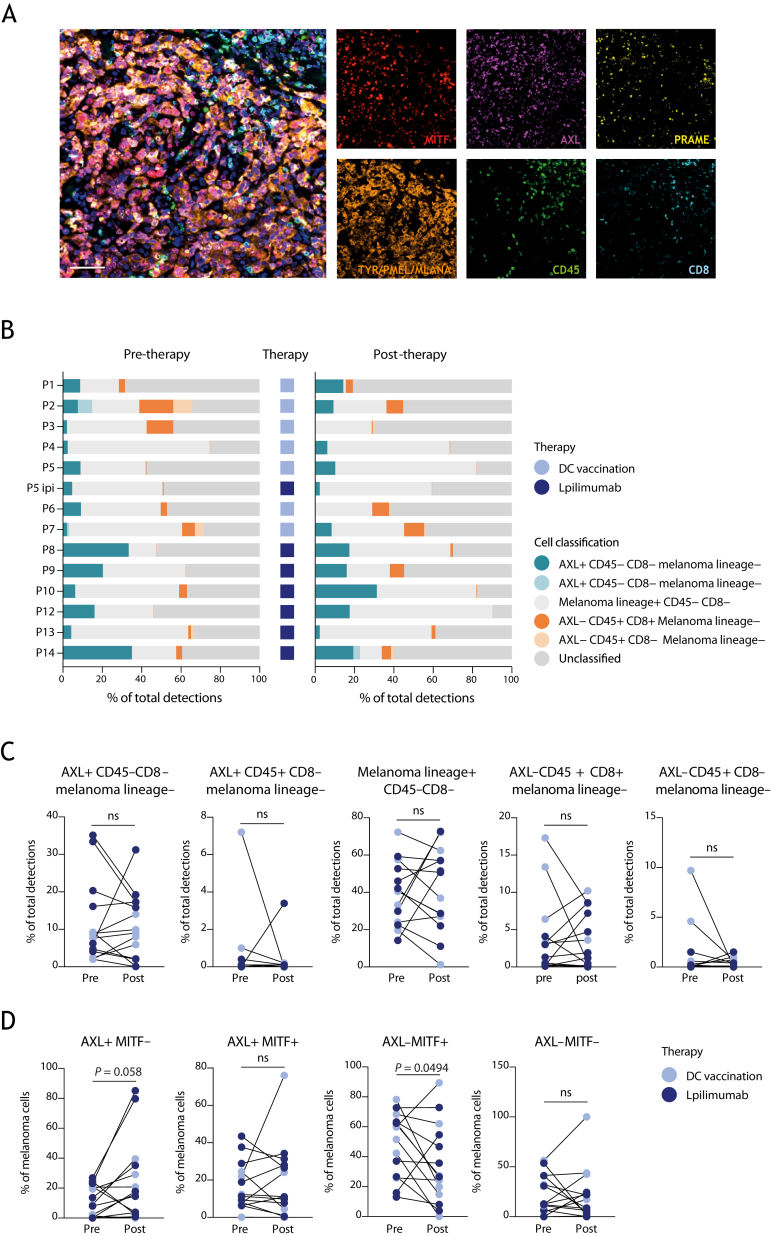
**Multiplex IF analysis of differential response of melanoma subpopulations to immunotherapy.** (A) Multiplex IF staining for MITF (red), AXL (purple), PRAME (yellow), the melanocyte differentiation antigens TYR, PMEL and MLANA (orange), CD45 (green), CD8 (cyan) and nuclei (blue). Scale bar, 25 μm. (B) Stacked bar chart showing the relative presence of different cell types before and after therapy and the type of therapy given (DC vaccination or ipilimumab) for each individual patient. Cell classifications are indicated in the graph. (C) Before–after plot showing the percentage of AXL+ CD45− melanoma lineage−, AXL+ CD45+ melanoma lineage−, melanoma lineage+, AXL− CD45+ CD8+ and AXL− CD45+ CD8− cells as a percentage of the total detected cells pre- and post-treatment. (D) Before–after plot showing the percentage of AXL+ MITF−, AXL+ MITF+, AXL− MITF+ and AXL− MITF− melanoma cells as a percentage of the total of melanoma cells pre- and post-treatment. DC vaccination is shown in lilac, and ipilimumab treatment is shown in purple. Paired *t*-test or Wilcoxon matched-pairs signed rank test significant as indicated; *P* < 0.05 is considered significant. Mean ± SEM. DC, dendritic cell; IF, immunofluorescence; ns, not significant; SEM, standard error of the mean.

To investigate the abundance of the various melanoma phenotypes and to validate the aforementioned subsets at the protein level, we carried out mIF staining. Besides analysis of AXL and MITF, PRAME, MLANA, TYR and PMEL were included as these were identified as differentially expressed from *in silico* analysis of the Tirosh et al.^9^ dataset. Moreover, including melanocyte differentiation antigens (TYR, PMEL, MLANA) helps to distinguish differentiated cells from dedifferentiated cells, while PRAME gives information on metastatic potential.^33^ Cells expressing at least one melanoma lineage marker (MITF, PRAME or the melanocyte differentiation antigens) and negative for CD45 were considered melanoma cells. Concomitantly, CD45 and CD8 were included to decipher CD8+ T-cell infiltration, indicating the level of immunological ‘pressure’ within the tumor tissue. AXL is both expressed by tumor and a variety of stromal cells, including CAFs.^34^ We explored this versatile expression in the context of melanoma in a previously published scRNAseq melanoma dataset,^26^ which revealed that MITF is specifically expressed in tumor cells, whereas AXL expression is highest in CAFs and tumor-associated macrophages (TAMs) (Supplementary Figure S3A-E, available at https://doi.org/10.1016/j.iotech.2024.101009). Consistent with the negative correlation between the AXL and MITF transcriptional programs in melanoma,^9^ we observed an inverse expression pattern between AXL and MITF. While MITF expression strongly and positively correlated with expression of the malignant cell signature, AXL displayed a negative correlation with this signature (Supplementary Figure S3F, available at https://doi.org/10.1016/j.iotech.2024.101009). AXL expression, on the other hand, positively correlated with gene signature expression of CAFs and macrophages (Supplementary Figure S3F, available at https://doi.org/10.1016/j.iotech.2024.101009). Our NanoString data (Figure 1A and B) confirmed these differential expression patterns for AXL and MITF (Supplementary Figure S3G, available at https://doi.org/10.1016/j.iotech.2024.101009). Based on these data, we defined AXL+ CD45− CD8− melanoma lineage− cells as potential CAFs, and AXL+ CD45+ CD8− melanoma lineage− cells as potential macrophages. In addition, an AXL+ CD45− CD8− melanoma lineage+ subpopulation of putative dedifferentiated melanoma cells was also detectable (Figure 3B and C).

It was feasible to detect the selected panel of markers by mIF (Figure 3A). To assess the clinical response of these subtypes to immunotherapy, we investigated paired biopsy specimens of patients undergoing DC vaccination or ipilimumab treatment by mIF (Figure 3B). Representative images pre- and post-treatment biopsies from four patients are shown in Supplementary Figure S4A, available at https://doi.org/10.1016/j.iotech.2024.101009. These images illustrate the co-occurrence of the distinct phenotypic cells. To explore if the different cell types were changing upon immunotherapy, we quantified the abundance of every cell subset pre- and post-therapy for every individual patient (Figure 3B and C). None of the cell types significantly changed upon treatment when related to the total number of cells detected in the tissue (Figure 3C). Nonetheless, despite large interpatient variability (Supplementary Figure S4, available at https://doi.org/10.1016/j.iotech.2024.101009) characterizing the changes of the four phenotypic melanoma cell subsets within the melanoma cell population, we observed a significant decrease in AXL− MITF+ melanoma cells (*P* = 0.0494) and a trend toward an increase in AXL+ MITF− melanoma cells upon treatment (*P* = 0.0580, Figure 3D). The latter was regardless of the type of therapy (Supplementary Figure S5, available at https://doi.org/10.1016/j.iotech.2024.101009), although we observed an increase in AXL+ MITF− cells in five of seven patients treated with ipilimumab (Supplementary Figure S5, available at https://doi.org/10.1016/j.iotech.2024.101009). These data demonstrate that despite the large variety in phenotypes and changes upon therapy, the percentage of AXL+ MITF− cells seems to increase in the majority (9 out of 14) of patients (Figure 3D).

Concluding, although it has been described that dedifferentiated AXL-expressing melanoma cells are considered to be resistant to targeted therapy using RAF and MEK inhibitors,^9^^,^^10^^,^^13^ our data suggest that AXL+ melanoma cells may also escape to immunotherapy, whereas MITF-expressing melanoma cells seem sensitive to immune-mediated killing.

### Melanoma heterogeneity in relation to immunological pressure

**Figure 4:**
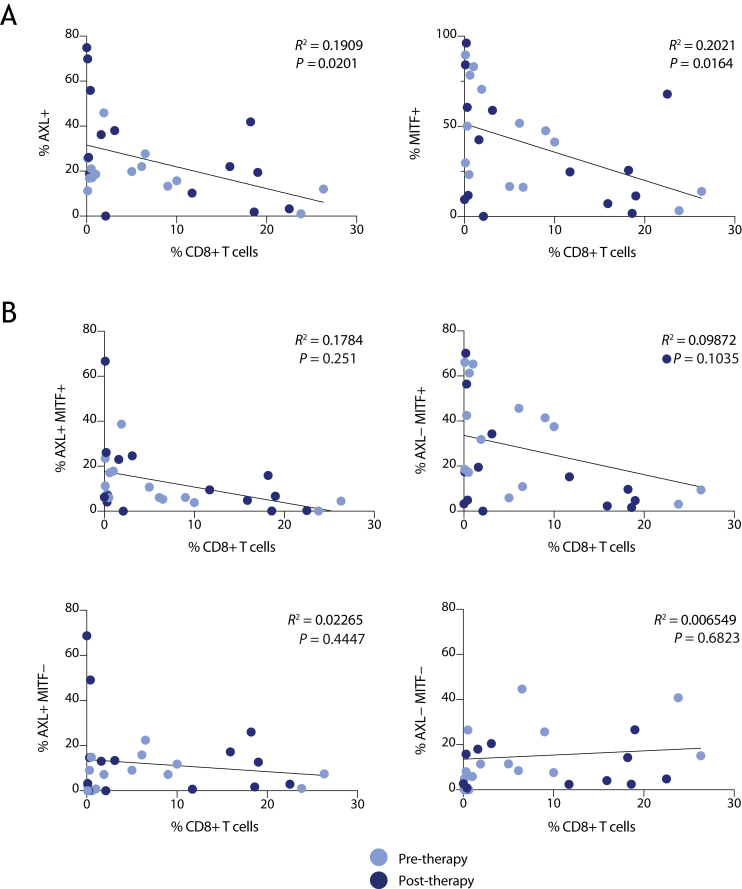
**Melanoma heterogeneity and the correlation with immunological pressure.** (A-B) Linear regression analysis (based on the mIF data) of the relative abundance of CD8+ T cells within a tumor and the relative abundance of AXL+ or MITF+ tumor cells (A), or of any of the four AXL/MITF melanoma subpopulations as a total of all classified cells (B). Pre-therapy samples are indicated in lilac, and post-therapy samples are indicated in purple. mIF, multiplex immunofluorescence.

Melanoma cell heterogeneity can be influenced by the presence of infiltrating immune cells and resulting immunological pressure. mIF did not reveal any correlation between the proportion of AXL or MITF melanoma cell phenotypes and the density of leukocyte infiltration, as identified by CD45 expression (not shown). As high infiltration of CD8+ T cells (intratumoral, stromal or invasive marginal), but not CD45+ cells, is predictive of immunotherapy outcome,^35^ we examined the presence of CD8+ CD45+ cells in these tumors in our mIF data. Abundance of AXL+ or MITF+ melanoma cells (Figure 4A), or AXL/MITF-double-positive cells (Figure 4B), showed a weak inverse correlation with the presence of CD8+ lymphocytes (Figure 4A). Interestingly, tumor tissue samples showing a higher percentage of CD8+ cells and lower abundance of MITF+ melanoma cells were taken post-treatment (Figure 4, purple dots), suggesting that DC vaccination or ipilimumab has enhanced immunoreactivity against these cells.

### Immunotherapy-induced changes in AXL+ MITF− cells do not correlate with improved survival

Next, we analyzed whether immunotherapy-induced changes in AXL+ cells (in the mIF data) correlated with patient survival in our cohort. The median survival of patients included in this study was 35.5 months (Table 1). Even though not significantly different, the median OS of patients treated with DC vaccination was higher compared with that of the ipilimumab-treated patients (46.5 versus 25.5 months) (Table 1). No significant difference in OS was observed between patients showing a decrease or increase in the proportion of any of the four melanoma subpopulations upon therapy (Supplementary Figure S6A, available at https://doi.org/10.1016/j.iotech.2024.101009). Similarly, a high proportion of MITF+ cells at baseline (Supplementary Figure S6B, available at https://doi.org/10.1016/j.iotech.2024.101009), irrespective of AXL status, as well as of any of the four AXL/MITF melanoma cell subpopulations (Supplementary Figure S6C, available at https://doi.org/10.1016/j.iotech.2024.101009), did not significantly affect the survival probability. However, patients with a high proportion of AXL+ melanoma cells (regardless of MITF expression) before therapy show a trend toward worse survival compared with patients with low pre-treatment AXL+ levels (Supplementary Figure S6D, available at https://doi.org/10.1016/j.iotech.2024.101009).

## Discussion

In this study, we observed a marked tumor heterogeneity among metastatic melanoma patients before immunotherapy. Although a clear association of any of the GOBPSlim terms or immune signatures with therapy response or survival was not observed, we identified the presence of previously described AXL+ and MITF+ subpopulations, both at the mRNA and protein levels. Our data demonstrate that the proportion of MITF+ melanoma cells is significantly decreased upon immunotherapy, while AXL+ melanoma cells seem to be more resistant. We also found a negative correlation between the proportion of MITF+ melanoma cells and CD8+ T-cell infiltration in the tumor tissue.

Based on transcriptomic data, the AXL and MITF subpopulations have been identified in melanoma and play a major role in tumor heterogeneity, as shown in our and other’s data.^9^^,^^36^ We here show that these melanoma subpopulations can also be detected at the protein level *in situ* using mIF. Although AXL+ cells are in general considered to be cancer cells, our analysis clearly revealed that AXL expression is highest in CAFs and TAMs. This is consistent with the earlier observation by Tirosh et al. which mentioned that the AXL program is expressed both in melanoma cells and CAFs. Future analysis on the role of AXL in the tumor microenvironment should therefore discriminate between the different cell types expressing AXL.

Emerging data from the literature show that AXL+ tumor cells contribute to increased resistance to targeted therapy, including mitogen-activated protein kinases inhibitors.^9^^,^^10^^,^^13^ More recent pre-clinical studies suggest that AXL melanoma subpopulations may also be more resistant to immunotherapy, as AXL targeting enhances the response of immune checkpoint blockers in lung cancer, melanoma^37^ and breast cancer^38^ models. Our observation of lower proportions of MITF+ melanoma cells in patients’ metastatic lesions taken after immunotherapy compared to pre-therapy supports the hypothesis of a more efficient targeting of these cells by therapy-induced immune response, while AXL+ melanoma cells may be more resistant. Multiple mechanisms may cause this differential response to immunotherapy. For example, MITF+ cells are more differentiated, expressing more melanocyte differentiation antigens (e.g. TYR, PMEL, MLANA) as well as HLA class I molecules making these cells more immunogenic, whereas AXL+ cells are likely more dedifferentiated and therefore less immunogenic.^7^^,^^39^ Alternatively, T-cell-secreted pro-inflammatory cytokines have been reported to promote dedifferentiation of melanoma cells by suppressing MITF expression,^40^ explaining why more infiltrated tumors have reduced numbers of MITF+ cells. Furthermore, emerging evidence suggests that AXL may also be actively involved in immune escape, through reduced HLA class I expression, and enhanced expression of several immunosuppressive cytokines, and programmed death-ligand 1.^41^ These cellular mechanisms may also (partially) explain the observation that the immunological pressure is most significantly associated with MITF+ cells. The fact that AXL+ melanoma cells are less immunogenic and therefore more resistant to immunotherapy was also suggested by Jerby-Arnon et al.^26^ They used scRNAseq to identify a melanoma resistance program in response to immune checkpoint inhibitors. Those resistant cells were reported to be slow-cycling and expressed a low MITF, dedifferentiated state, including *AXL* mRNA expression.^10^^,^^42^^,^^43^ Similarly, Pozniak et al. recently reported the role of TCF4-driven mesenchymal melanoma cells in immunotherapy resistance.^44^ Also, here the immune exclusion program did include repression of genes involved in antigen processing and presentation.^26^ In our study, we also observed a (moderate) increase in AXL+ MITF− cells in five out of seven patients treated with an ICI (ipilimumab). However, intrinsic resistance of AXL+ tumor cells did not reach significance in our cohort, due to the limited number of patients.

To date, resistance of AXL+ MITF− cells to DC vaccination has been largely unexplored. Our data demonstrate that four out of seven patients who were treated with DC vaccination show an increase in AXL+ MITF− cells. This observation suggests that AXL+ MITF− melanoma cells are also more resistant to DC vaccination, possibly due to reduced immunogenicity.

Data on tumor heterogeneity in melanoma and resistance to various immunotherapies are emerging, yet it remains critical to characterize tumor cell subpopulations underlying tumor heterogeneity in relation to therapy response in more detail. Our results suggest that MITF+ melanoma cells are efficiently targeted by immunotherapy, while AXL+ melanoma cells may be more resistant. This indicates an analogous sensitivity profile of MITF or AXL melanoma cells to both targeted and immunotherapy. It may also explain why important prognostic factors for metastatic melanoma, like performance status and lactate dehydrogenase levels, appear to be predictive of benefit from either of these therapeutic modalities.^45^ Our findings indicate the need of developing novel and alternative therapeutic strategies for targeting treatment-resistant, AXL+ melanoma subpopulations.

## References

[1] Quaglino P., Fava P., Tonella L. (2021). Treatment of advanced metastatic melanoma. Dermatol Pract Concept.

[2] Rauwerdink D.J.W., Molina G., Frederick D.T. (2020). Mixed response to immunotherapy in patients with metastatic melanoma. Ann Surg Oncol.

[3] Jager E., Ringhoffer M., Karbach J., Arand M., Oesch F., Knuth A. (1996). Inverse relationship of melanocyte differentiation antigen expression in melanoma tissues and CD8+ cytotoxic-T-cell responses: evidence for immunoselection of antigen-loss variants in vivo. Int J Cancer.

[4] Riker A., Cormier J., Panelli M. (1999). Immune selection after antigen-specific immunotherapy of melanoma. Surgery.

[5] Ohnmacht G.A., Wang E., Mocellin S. (2001). Short-term kinetics of tumor antigen expression in response to vaccination. J Immunol.

[6] Sade-Feldman M., Jiao Y.J., Chen J.H. (2017). Resistance to checkpoint blockade therapy through inactivation of antigen presentation. Nat Commun.

[7] Hoek K.S., Goding C.R. (2010). Cancer stem cells versus phenotype-switching in melanoma. Pigment Cell Melanoma Res.

[8] Schatton T., Schütte U., Frank N.Y. (2010). Modulation of T-cell activation by malignant melanoma initiating cells. Cancer Res.

[9] Tirosh I., Izar B., Prakadan S.M. (2016). Dissecting the multicellular ecosystem of metastatic melanoma by single-cell RNA-seq. Science.

[10] Muller J., Krijgsman O., Tsoi J. (2014). Low MITF/AXL ratio predicts early resistance to multiple targeted drugs in melanoma. Nat Commun.

[11] Rambow F., Marine J.C., Goding C.R. (2019). Melanoma plasticity and phenotypic diversity: therapeutic barriers and opportunities. Genes Dev.

[12] Flaherty K.T., Infante J.R., Daud A. (2012). Combined BRAF and MEK inhibition in melanoma with BRAF V600 mutations. N Engl J Med.

[13] Konieczkowski D.J., Johannessen C.M., Abudayyeh O. (2014). A melanoma cell state distinction influences sensitivity to MAPK pathway inhibitors. Cancer Discov.

[14] Spranger S., Bao R., Gajewski T.F. (2015). Melanoma-intrinsic beta-catenin signalling prevents anti-tumour immunity. Nature.

[15] Byers L.A., Diao L., Wang J. (2013). An epithelial-mesenchymal transition gene signature predicts resistance to EGFR and PI3K inhibitors and identifies Axl as a therapeutic target for overcoming EGFR inhibitor resistance. Clin Cancer Res.

[16] Hugo W., Shi H., Sun L. (2015). Non-genomic and immune evolution of melanoma acquiring MAPKi resistance. Cell.

[17] Haas L., Elewaut A., Gerard C.L. (2021). Acquired resistance to anti-MAPK targeted therapy confers an immune-evasive tumor microenvironment and cross-resistance to immunotherapy in melanoma. Nat Cancer.

[18] Lim S.Y., Shklovskaya E., Lee J.H. (2023). The molecular and functional landscape of resistance to immune checkpoint blockade in melanoma. Nat Commun.

[19] Yin T., Wang G., Wang L. (2024). Breaking NGF-TrkA immunosuppression in melanoma sensitizes immunotherapy for durable memory T cell protection. Nat Immunol.

[20] Thornton J., Chhabra G., Singh C.K. (2022). Mechanisms of immunotherapy resistance in cutaneous melanoma: recognizing a shapeshifter. Front Oncol.

[21] Peng W., Chen J.Q., Liu C. (2016). Loss of PTEN promotes resistance to T cell-mediated immunotherapy. Cancer Discov.

[22] Gopalakrishnan V., Spencer C.N., Nezi L. (2018). Gut microbiome modulates response to anti-PD-1 immunotherapy in melanoma patients. Science.

[23] Landsberg J., Kohlmeyer J., Renn M. (2012). Melanomas resist T-cell therapy through inflammation-induced reversible dedifferentiation. Nature.

[24] Miranda A., Hamilton P.T., Zhang A.W. (2019). Cancer stemness, intratumoral heterogeneity, and immune response across cancers. Proc Natl Acad Sci U S A.

[25] Bulgarelli J., Tazzari M., Granato A.M. (2019). Dendritic cell vaccination in metastatic melanoma turns “non-T cell inflamed” into “T-cell inflamed” tumors. Front Immunol.

[26] Jerby-Arnon L., Shah P., Cuoco M.S. (2018). A cancer cell program promotes T cell exclusion and resistance to checkpoint blockade. Cell.

[27] Stuart T., Butler A., Hoffman P. (2019). Comprehensive integration of single-cell data. Cell.

[28] Cancer Genome Atlas Network (2015). Genomic classification of cutaneous melanoma. Cell.

[29] Bankhead P., Loughrey M.B., Fernandez J.A. (2017). QuPath: open source software for digital pathology image analysis. Sci Rep.

[30] Kumpers C., Jokic M., Haase O. (2019). Immune cell infiltration of the primary tumor, not PD-L1 status, is associated with improved response to checkpoint inhibition in metastatic melanoma. Front Med (Lausanne).

[31] Goding C.R., Arnheiter H. (2019). MITF-the first 25 years. Genes Dev.

[32] Kress T.R., Sabo A., Amati B. (2015). MYC: connecting selective transcriptional control to global RNA production. Nat Rev Cancer.

[33] Cassalia F., Danese A., Tudurachi I. (2024). PRAME updated: diagnostic, prognostic, and therapeutic role in skin cancer. Int J Mol Sci.

[34] Tanaka M., Siemann D.W. (2020). Gas6/Axl signaling pathway in the tumor immune microenvironment. Cancers (Basel).

[35] Li F., Li C., Cai X. (2021). The association between CD8+ tumor-infiltrating lymphocytes and the clinical outcome of cancer immunotherapy: a systematic review and meta-analysis. EClinicalMedicine.

[36] Kim Y.S., Kim D., Park J., Chung Y.J. (2023). Single-cell RNA sequencing of a poorly metastatic melanoma cell line and its subclones with high lung and brain metastasis potential reveals gene expression signature of metastasis with prognostic implication. Exp Dermatol.

[37] Boshuizen J., Pencheva N., Krijgsman O. (2021). Cooperative targeting of immunotherapy-resistant melanoma and lung cancer by an AXL-targeting antibody-drug conjugate and immune checkpoint blockade. Cancer Res.

[38] Aguilera T.A., Rafat M., Castellini L. (2016). Reprogramming the immunological microenvironment through radiation and targeting Axl. Nat Commun.

[39] Schieven S.M., Traets J.J.H., Vliet A.V. (2023). The elongin BC complex negatively regulates AXL and marks a differentiated phenotype in melanoma. Mol Cancer Res.

[40] Riesenberg S., Groetchen A., Siddaway R. (2015). MITF and c-Jun antagonism interconnects melanoma dedifferentiation with pro-inflammatory cytokine responsiveness and myeloid cell recruitment. Nat Commun.

[41] Son H.Y., Jeong H.K. (2021). Immune evasion mechanism and AXL. Front Oncol.

[42] Ahn A., Chatterjee A., Eccles M.R. (2017). The slow cycling phenotype: a growing problem for treatment resistance in melanoma. Mol Cancer Ther.

[43] Perego M., Maurer M., Wang J.X. (2018). A slow-cycling subpopulation of melanoma cells with highly invasive properties. Oncogene.

[44] Pozniak J., Pedri D., Landeloos E. (2024). A TCF4-dependent gene regulatory network confers resistance to immunotherapy in melanoma. Cell.

[45] Ascierto P.A., Ribas A., Larkin J. (2020). Impact of initial treatment and prognostic factors on postprogression survival in BRAF-mutated metastatic melanoma treated with dacarbazine or vemurafenib +/− cobimetinib: a pooled analysis of four clinical trials. J Transl Med.

