# Supplementary figures and images for: Changes in AXL and/or MITF melanoma subpopulations in patients receiving immunotherapy
Source: Immunooncol Technol. 2024 Nov 15;24:101009. doi: 10.1016/j.iotech.2024.101009 (PMC11652950; doi:10.1016/j.iotech.2024.101009)

Supplementary Figure S1

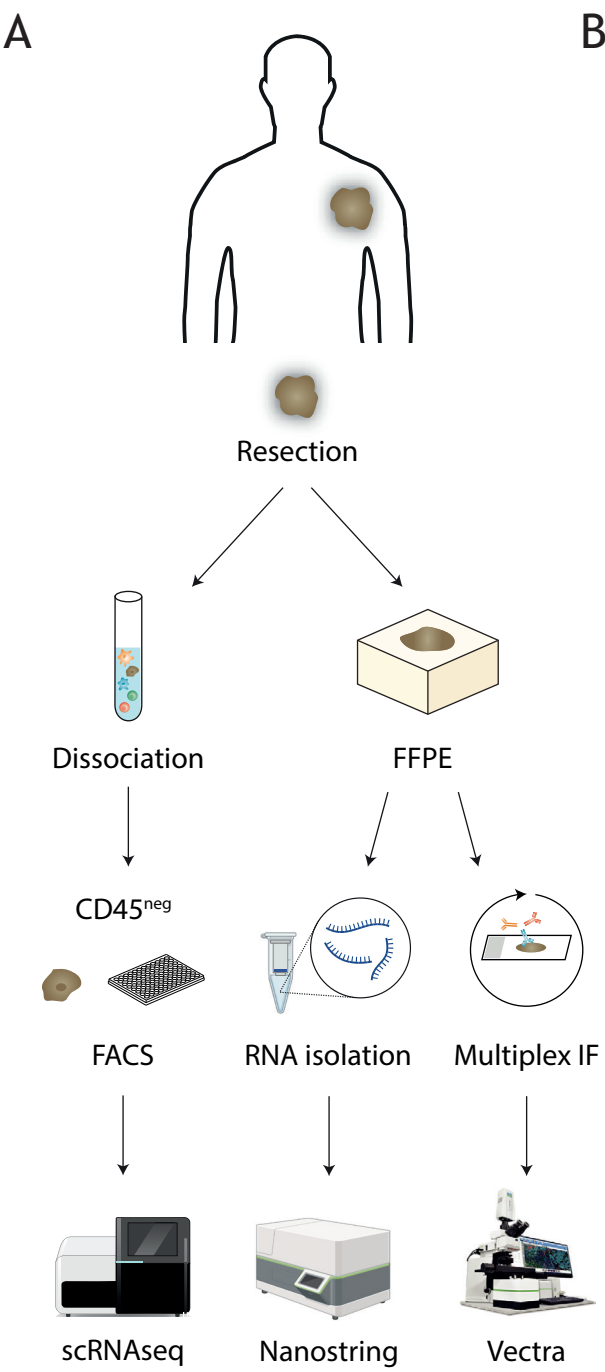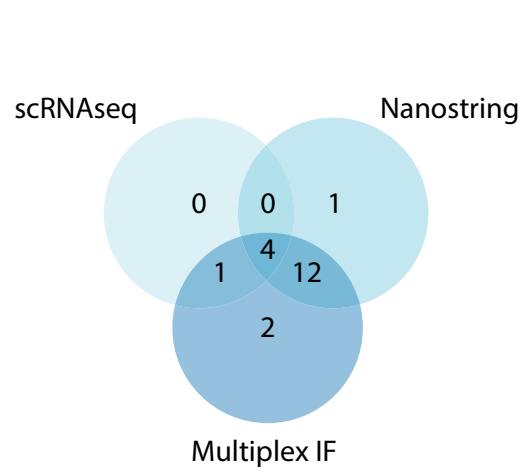

Supplement: Supplementary Figure S1 [file mmc1.pdf]

Supplementary Figure S2

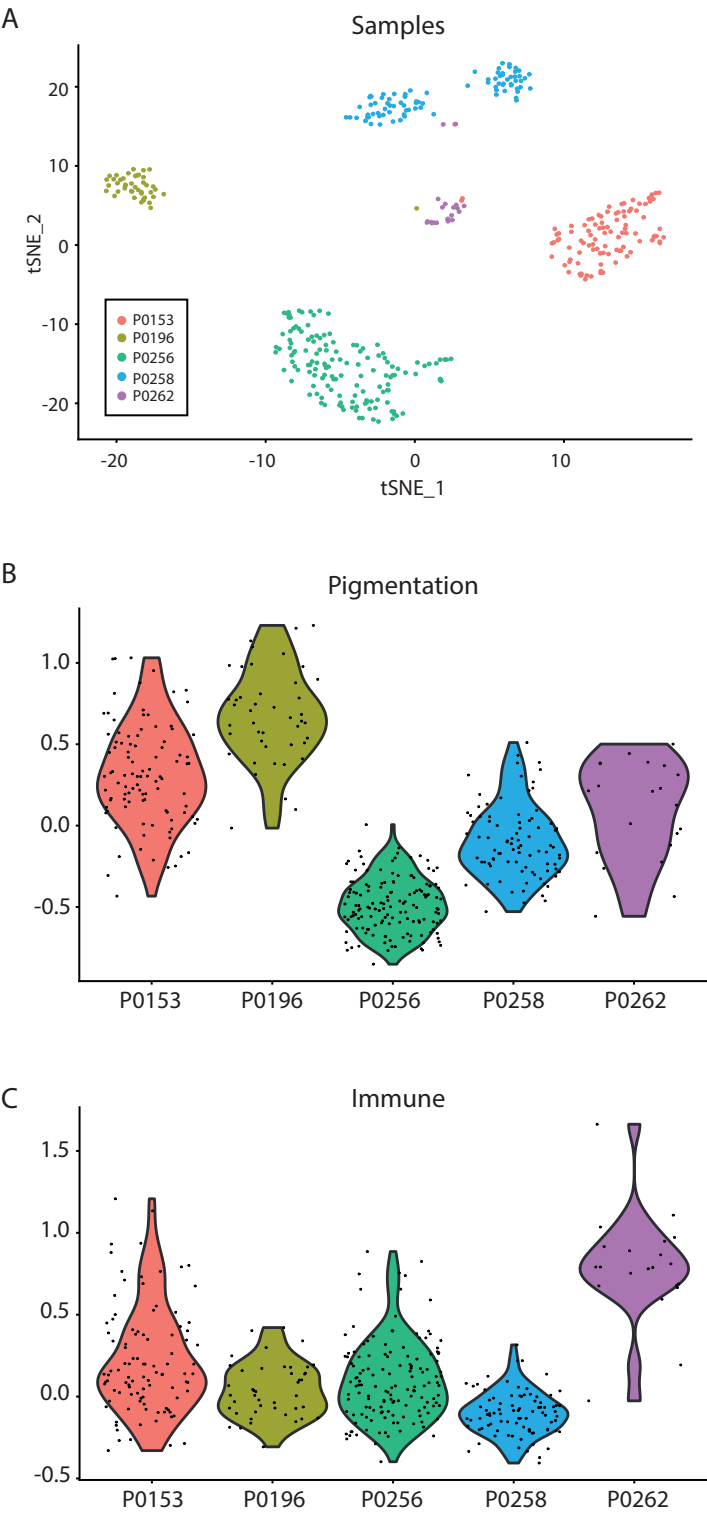

Supplement: Supplementary Figure S2 [file mmc2.pdf]

Supplementary Figure S3

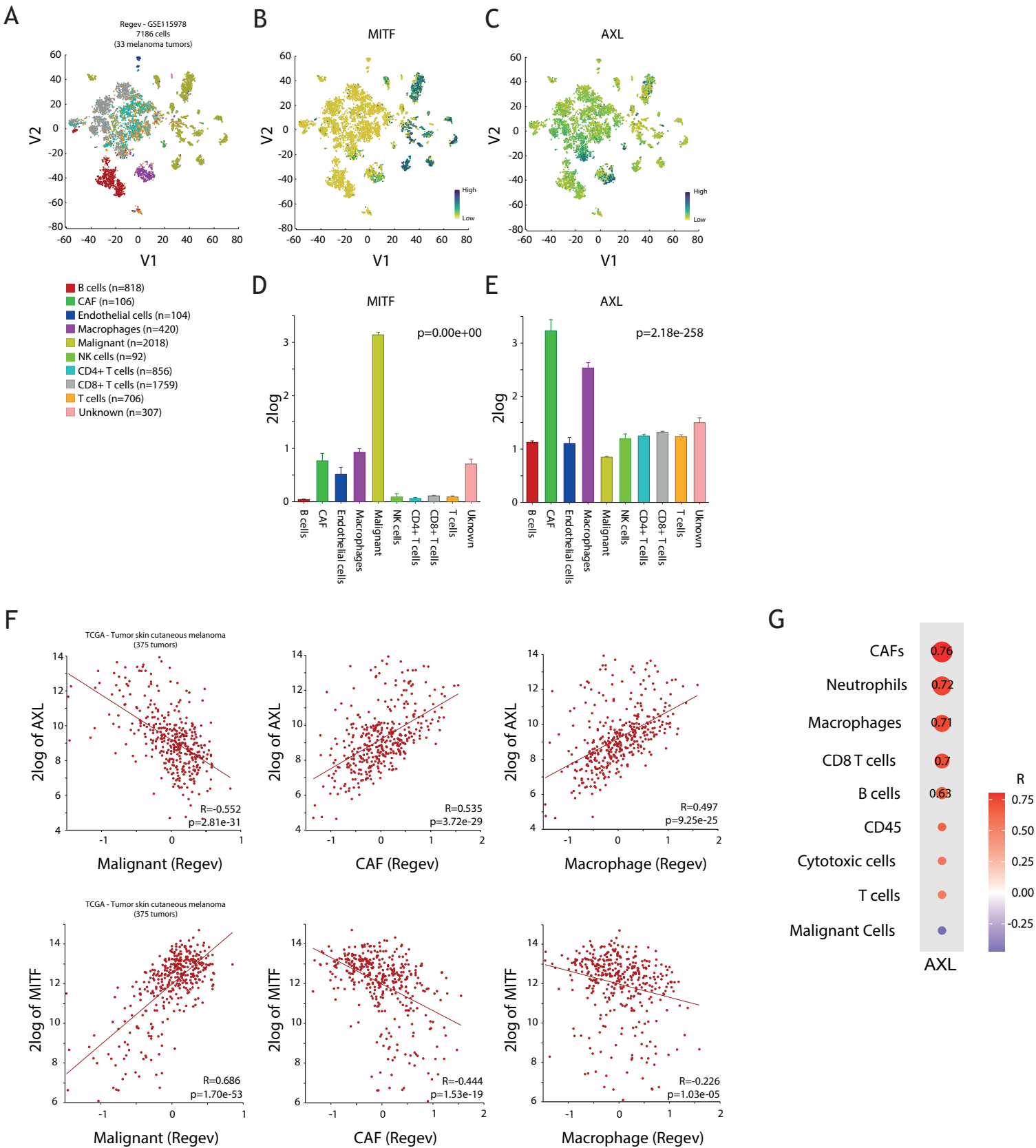

Supplement: Supplementary Figure S3 [file mmc3.pdf]

Supplementary Figure S4

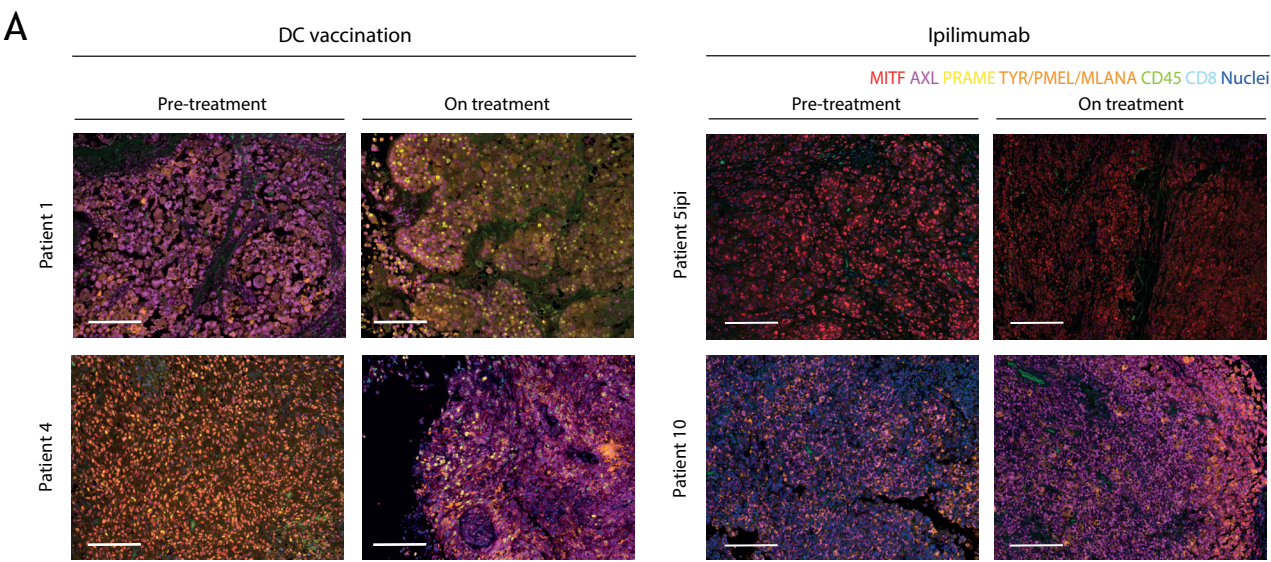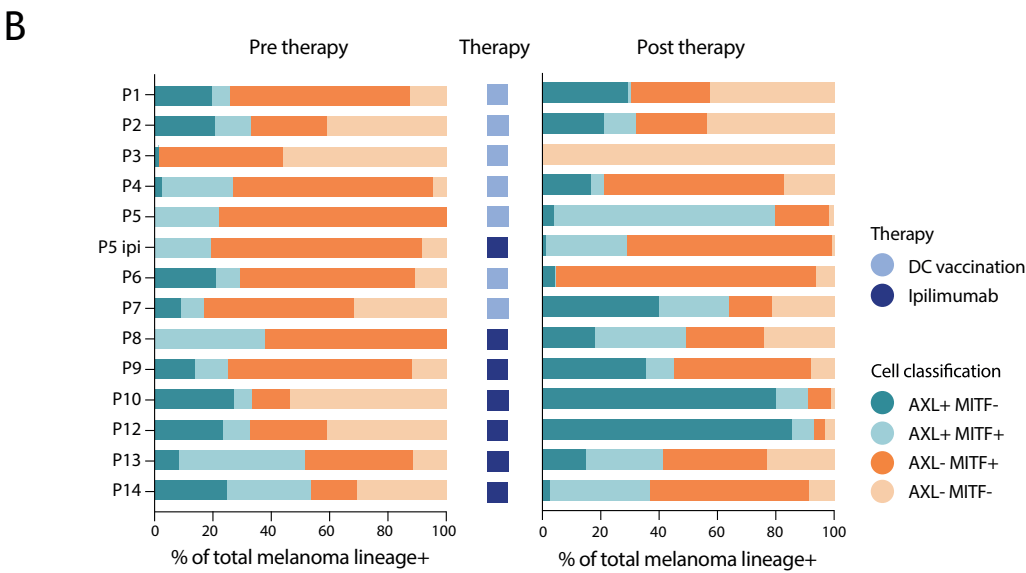

Supplement: Supplementary Figure S4 [file mmc4.pdf]

Supplementary Figure S5

DC vaccination

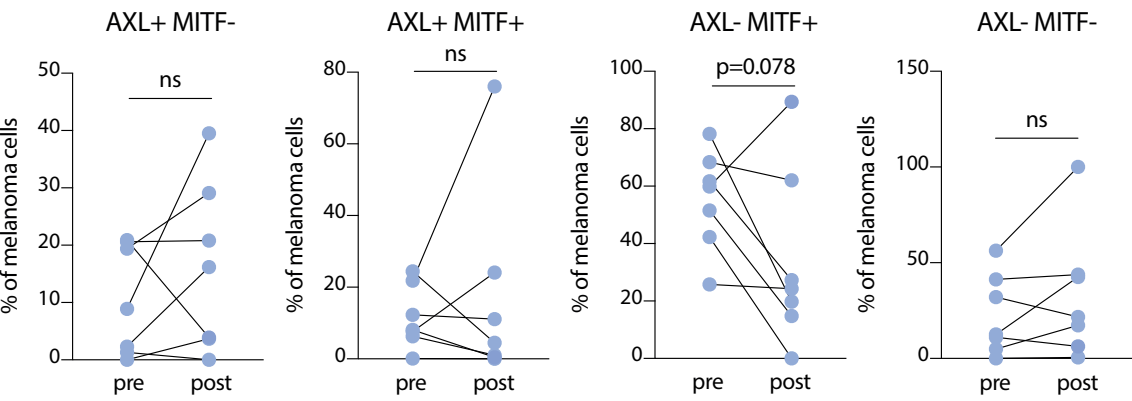

Ipilimumab

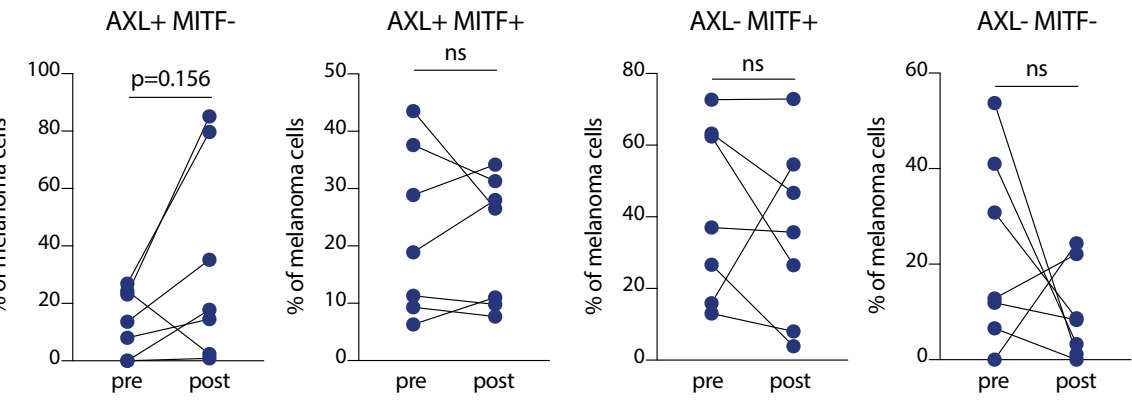

Therapy

- DC vaccination
- Ipilimumab

Supplement: Supplementary Figure S5 [file mmc5.pdf]

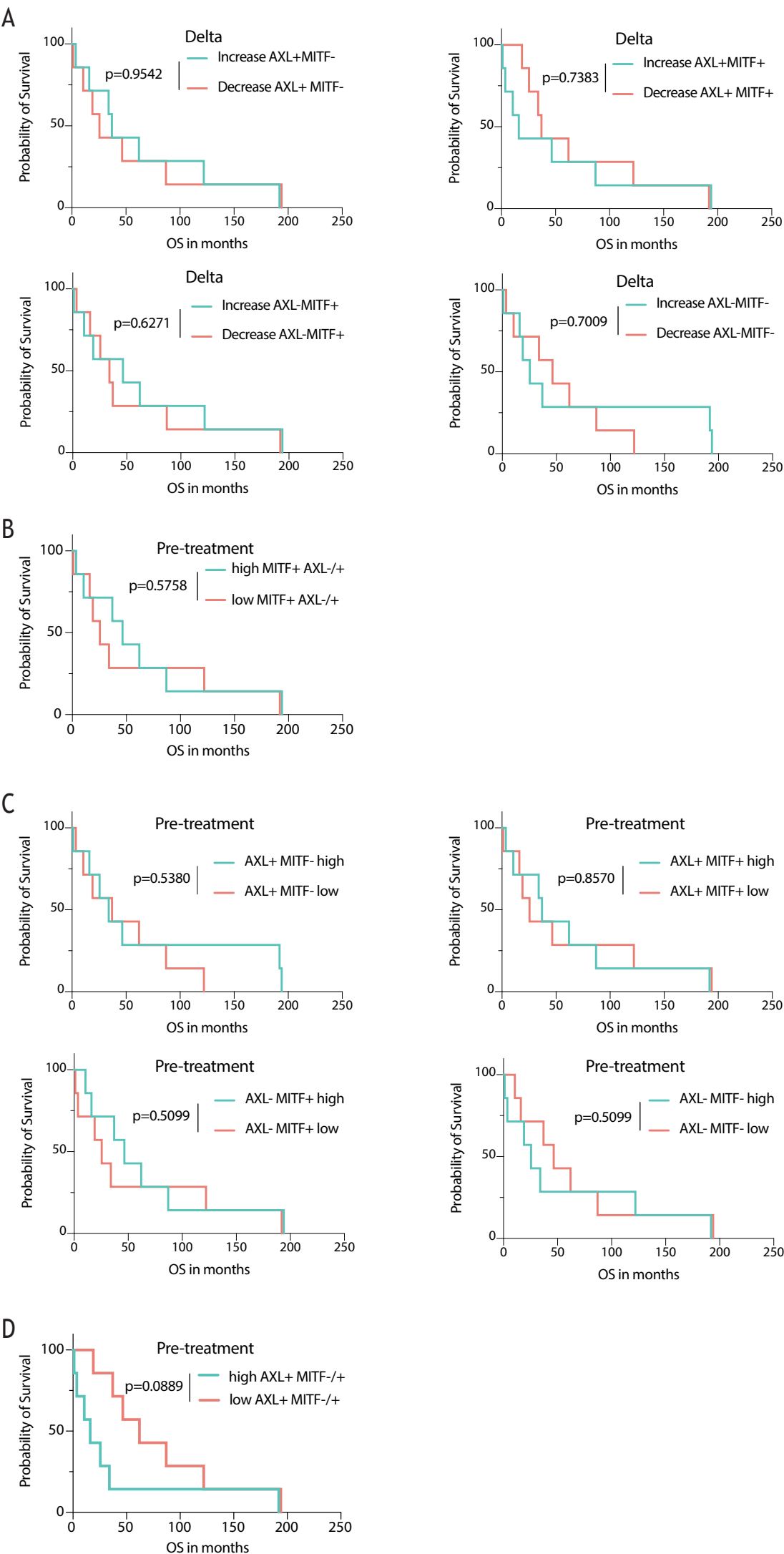

Supplement: Supplementary Figure S6 [file mmc6.pdf]
