# Supplementary material for: Changes in AXL and/or MITF melanoma subpopulations in patients receiving immunotherapy
Source: Immunooncol Technol. 2024 Nov 15;24:101009. doi: 10.1016/j.iotech.2024.101009 (PMC11652950; doi:10.1016/j.iotech.2024.101009)
Supplement: Supplementary Table S1 [file mmc7.docx]

**Supplementary Table S1. Number of sequenced cells classified to each cell type in each tumor**

| **Tumor** | **AXL^high^ MITF^low^** | **AXL^high^ MITF^high^** | **AXL^low^ MITF^high^** | **AXL^low^ MITF^low^** | **Total** |
| --- | --- | --- | --- | --- | --- |
| P0153 | 12 | 54 | 10 | 24 | 100 |
| P0196 | 0 | 1 | 41 | 2 | 44 |
| P0256 | 68 | 39 | 0 | 36 | 143 |
| P0258 | 0 | 1 | 3 | 83 | 87 |
| P0262 | 0 | 15 | 1 | 5 | 21 |
