# Supplementary material for: Changes in AXL and/or MITF melanoma subpopulations in patients receiving immunotherapy
Source: Immunooncol Technol. 2024 Nov 15;24:101009. doi: 10.1016/j.iotech.2024.101009 (PMC11652950; doi:10.1016/j.iotech.2024.101009)
Supplement: Supplementary materials [file mmc10.docx]

**Supplementary data**

**Supplementary Figure S1. Workflow of the analyses performed**

(**A**) Overview of workflow. FFPE, formalin fixed paraffin embedded; FACS, fluorescence-activated cell sorting; IF, immunofluorescence; scRNAseq, single-cell RNA sequencing. (**B**) Venn diagram showing the overlap of patients between the different analyses.

**Supplementary Figure S2.**

**(A)** T-stochastic neighbor embedding plot (t-SNE) of 395 cells from 5 fresh samples (Table 1) analyzed by single-cell RNA sequencing. Each sample is presented in a different color (as indicated in the figure). (**B**) Analysis of the pigmentation geneset signature, or the immune geneset signature (**C**) in the individual samples. The Kruskal-Wallis test shows significant differential expression of the pigmentation (2.50E-49) or the immune- (5.71E-10) signature among the samples. Applied gene signatures are shown in Supplementary Table S2.

**Supplementary figure S3. scRNAseq melanoma dataset analysis**

**(A**) T-stochastic neighbor embedding plot (t-SNE, perplexity = 50) using single-cell RNAseq analysis of 7186 cells from 33 melanoma tumors.^26^ Cell types underneath graph; CAF (cancer-associated fibroblasts), NK (natural killer cells). MITF (**B**) and AXL (**C**) expression in the t-SNE plot from (A). Bar graphs related to (B, C) showing MITF (**D**) and AXL (**E**) expression in the different cell types. P-value indicates One Way Analysis of variance (ANOVA). (**F**) XY-plots showing the correlation (R, correlation coefficient) and statistical significance (p-value) of AXL or MITF mRNA expression with expression of indicated geneset signatures in the TCGA melanoma data. (**G**) Correlation between AXL mRNA with melanoma and immune cell gene signatures in pre-immunotherapy melanoma tumor samples as identified by IO360 nanostring gene assays.

**Supplementary figure S4. Phenotypic heterogeneity between melanoma patients**

(**A**) Multiplex IF staining on melanoma metastases from patients treated with DC vaccination (patient 1 and 4) or ipilimumab (patient 5ipi and 10): pre-treatment (left) and on treatment (right). The color of each of the markers is indicated. Scale bar, 50 µm. (**B**) Stacked bar chart showing the relative presence of different melanoma subsets prior to and after therapy and the type of therapy given (DC vaccination or ipilimumab) for each individual patient.

**Supplementary figure S5. MITF expression and the correlation with immunological pressure**

Before-after plot showing the percentage of AXL+ MITF-, AXL+ MITF+, AXL- MITF+, and AXL- MITF- melanoma cells as a percentage of the total of melanoma cells pre and post treatment based on the mIF data. Similar as in figure 3D, but now data is separated for each therapy: DC vaccination is shown in lilac, ipilimumab treatment is shown in purple. Paired t test or Wilcoxon matched-pairs signed rank test significant as indicated; ns, not significant, P < 0.05 is considered significant. Mean ± SEM.

**Supplementary figure S6. Low pre-treatment levels of AXL+ MITF-/+ cells trend towards a correlation with better clinical outcome**

(**A**) Kaplan-Meier analysis showing the overall survival (OS) of metastatic melanoma patients, comparing patients with an increase (cyan) or a decrease (red) in any of the AXL/MITF melanoma cell subpopulations during therapy. All figures (S5A-D) are based on expression levels as determined by the mIF analysis. A log-rank test was applied to assess significance. (**B**) similar as in (A) but for MITF high or low melanoma cells, irrespective of the levels of AXL (AXL+/-). High- or low expression (S4B-D) was determined based on the mIF data defining e.g. MITF high-expressing cells as cells with above median expression of MITF and vice versa. (**C**) Kaplan-Meier analysis showing the overall survival (OS) of metastatic melanoma patients, comparing patients showing a high (cyan) or low (red) pre-treatment levels of any of the AXL/MITF melanoma subpopulations. A log-rank test was applied to assess significance. (**D**) Kaplan-Meier analysis showing the overall survival (OS) of metastatic melanoma patients, comparing patients showing a high (cyan) or low (red) pre-treatment levels of AXL+ MITF-/+ cells A log-rank test was applied to assess significance.
